# Gastrointestinal risk factors and patient‐reported outcomes of ankylosing spondylitis in Korea

**DOI:** 10.1111/1756-185X.13758

**Published:** 2019-12-29

**Authors:** Sang‐Hoon Lee, Yong‐wook Park, Jung‐Yoon Choe, Kichul Shin, Seong‐Ryul Kwon, Jin‐Hye Cha, Young‐Joo Kim, Juneyoung Lee, Tae‐Hwan Kim

**Affiliations:** ^1^ Department of Rheumatology, Hospital at Gangdong School of Medicine Kyung Hee University Seoul South Korea; ^2^ Department of Rheumatology Chonnam National University Hospital Gwangju Korea; ^3^ Department of Rheumatology Catholic University of Daegu School of Medicine Daegu Korea; ^4^ Department of Rheumatology SMG‐SNU Boramae Medical Center Seoul Korea; ^5^ Department of Rheumatology Inha University Hospital Incheon Korea; ^6^ Pfizer Pharmaceuticals Korea Limited Seoul Korea; ^7^ Department of Biostatistics Korea University College of Medicine Seoul Korea; ^8^ Department of Rheumatology The Hanyang University Hospital for Rheumatic Diseases Seoul Korea

**Keywords:** ankylosing spondylitis, gastrointestinal risk, NSAID, patient‐reported outcomes, quality of life

## Abstract

**Aim:**

This study examined the degree of gastrointestinal (GI) risk and patient‐reported outcomes including GI‐related symptoms, adherence to non‐steroidal anti‐inflammatory drugs (NSAIDs), disease activity and quality of life (QoL) in patients with ankylosing spondylitis (AS).

**Methods:**

Cross‐sectional, observational study conducted at six nationwide, university‐based hospitals of Korea. AS patients treated with NSAIDs for at least 2 weeks were included between March and September 2016. Demographic and clinical data were gathered through a medical chart review and patient survey. GI risk was estimated using Standardized Calculator of Risk for Events (SCORE). NSAIDs adherence was investigated with Morisky Medication Adherence Scale‐8 (MMAS‐8). Disease activity and QoL were examined with Bath Ankylosing Spondylitis Disease Activity Index (BASDAI) and EuroQol‐3L (EQ‐5D, EQ‐visual analog scale [EQ‐VAS]), respectively. Path analysis was implemented to estimate pathways of GI risk, GI symptoms and NSAIDs adherence to QoL.

**Results:**

A total of 596 patients (age: 38.9 ± 12.6 years, male: 82.1%) participated in the study, of which 33.2% experienced GI symptoms during NSAID treatment, and 34.2% of them showed ongoing GI symptoms upon enrollment. According to SCORE, 37.1% of patients showed moderate to very high GI risk. No patient showed high adherence according to MMAS‐8, so 55.3% of patients with moderate adherence were considered adherent. BASDAI and QoL of the total patients were 3.5 ± 2.0, 0.6 ± 0.3 (EQ‐5D), and 67.4 ± 19.8 (EQ‐VAS), respectively. From path analyses, higher GI risk significantly lowered QoL.

**Conclusion:**

This study suggests timely therapeutic strategies should be implemented to manage GI risk during NSAID treatment in order to effectively manage AS.

## 1 INTRODUCTION

Ankylosing spondylitis (AS) is an inflammatory arthritis affecting approximately fewer than 1% of the worldwide population.^1^{Braun, 1998 #1662} According to the 2016 update of the Assessment in Ankylosing Spondylitis International Society (ASAS) and European League Against Rheumatism (EULAR) management recommendations for AS, non‐steroidal anti‐inflammatory drugs (NSAIDs) are recommended as a first‐line treatment option up to its maximum dose in AS patients with pain and stiffness.^2^ In addition, NSAIDs were described as the key drugs for the effective management of AS.^3^ Despite the clinical usefulness of NSAIDs, they should be carefully selected and prescribed as they often lead to an increase in gastrointestinal (GI) risk that may later lead to the development of GI‐related complications.^4^, ^5^, ^6^ AS patients commonly suffer from an inflamed GI tract.^7^ Gastrointestinal side effects have been associated with NSAID use, which may be minor, such as nausea, dyspepsia, anorexia, and abdominal pain, or life‐threatening complications such as GI bleeding and perforation.^8^ This gastrointestinal risk among NSAID users is known to differ as per demographic and clinical characteristics of each individual patient,^9^ and known factors include long‐term use and/or high doses of NSAIDs (≥3 months), old age (≥65 years), heavy smoking, heavy drinking, current health status, co‐morbidity (cardiovascular, renal and liver diseases, diabetes, hypertension), diagnosis of rheumatoid arthritis, aspirin use, anticoagulant use, corticosteroid use, use of selective serotonin reuptake inhibitor, *Helicobacter pylori* infection, history of GI symptoms, and history of hospitalization due to GI complications.^10^ Therefore, a systematic treatment approach which considers each patient‐specific feature should be implemented to minimize GI risk while on a NSAIDs prescription.

Patients’ behaviors toward NSAIDs intake need to be monitored. AS is a chronic disease which requires life‐long treatment after onset; however, medication adherence of chronic diseases often decreases over time which could compromise the efficacy of NSAIDs.^11^ In a systematic review, adherence rate of NSAIDs users was reported between 30% to 65%.^12^ According to a double‐blind and randomized controlled trial including 140 AS patients, 32% of all patients reported missing 2‐10 days of taking NSAIDs medication.^13^ Along with clinical considerations for effective AS management, the assessment of patient‐reported outcomes (PROs) such as quality of life (QoL) and functional status is an important aspect that should be taken into account as they impact the daily lives of patients. The 2016 update of ASAS/EULAR guidelines clearly state that the primary objective of AS treatment should focus on the maximization of long‐term QoL.^2^ Previous study findings have demonstrated that the QoL of AS patients was severely impaired and lowered than that of the general population.^14^, ^15^, ^16^ In addition, the assessment of disease activity is 1 of the widely used PROs to evaluate functional status. Moreover, several studies have shown that QoL and functional status are closed related with each other.^17^, ^18^, ^19^

According to previous findings, GI risk, NSAID adherence and PROs including disease activity and QoL showed significant associations with each other. GI‐related disorders had negative impacts on NSAID adherence and QoL.^20^, ^21^ However, their complex inter‐relationships have been merely studied. Therefore, this study was primarily designed to understand the degree of GI risk and PROs including GI‐related symptoms, NSAIDs adherence, disease activity, and QoL, in AS patients. We have further evaluated their inter‐relationships by drawing potential paths to QoL.

## 2 METHODS

This was a cross‐sectional, observational study conducted at six nationwide, university‐based hospitals of Korea. Data were collected through medical chart review and patients’ self‐administered questionnaires between March and September, 2016. An informed written consent was signed by all patients prior to their enrollment, and all participating hospitals obtained approval from an Institutional Review Board prior to conducting the study. All procedures in this study have been performed in accordance with the ethical standards of the institution and/or national research committee and with the 1964 Declaration of Helsinki and its later amendments or comparable ethical standards.

### 2.1 Data collection

Patients who were diagnosed with AS according to the 1984 Modified New York criteria,^22^ and current NSAID users who were treated for at least 2 weeks were considered eligible. Those who were prescribed with NSAIDs as pro re nata, concurrently participating in other drug‐controlled studies, in severe/insecure clinical/mental conditions, or confirmed ineligible by the physicians’ discretion, were excluded. Patients who met all inclusion criteria were asked to participate on their regular visit to the participating hospitals. The study consecutively enrolled patients and collected data on demographic and clinical features, treatment patterns, NSAIDs adherence, disease activity, and QoL.

### 2.2 Gastrointestinal risk level: Standardized Calculator of Risk for Events (SCORE)

Gastrointestinal risk was calculated using SCORE.^23^ SCORE was based on six predictors: age, current health status, diagnosis of rheumatoid arthritis (RA) which was confirmed by predominant peripheral joint arthritis in this study, duration of corticosteroid use, GI symptoms such as bleedings or ulcers, and hospitalization history due to GI‐related symptoms or complications such as heartburn, stomach pain, nausea, vomiting while taking NSAIDs. The six predictors consisted of yes/no and multiple‐choice questions and patients earned a certain score based on the degree of their response to each predictor. A total of the SCORE value ranged from 0 (possible minimum SCORE value) to 37 (possible maximum SCORE value) and was calculated for each patient by adding up all points earned from the six predictors. Patients were further stratified into four risk groups according to the total of their SCORE value. A SCORE value of 10 or less indicated that their risk was low; 11‐15 points indicated a moderate risk; 16‐20 points indicated a high risk; and more than 20 points indicated a very high risk.

### 2.3 Medication adherence: Morisky Medication Adherence Scale‐8 (MMAS‐8) and NSAID intake rate

The MMAS‐eight was used to estimate NSAIDs adherence. It consists of eight items, with scores ranging from 0 to 8 according to the scoring given to each item.^24^ The level of adherence was determined as: high adherence = 8, moderate adherence = 6‐7, low adherence <6. The patients with high or moderate adherence were defined as NSAID adherent, and those showing low adherence were categorized as non‐adherent. In addition to MMAS‐8, NSAIDs intake rate of each patient was calculated based on the formula below.^25^

NSAID use by means of the ASAS‐NSAID score (0‐100):$\begin{array}{c} \text{Index}\;\text{of}\;\text{NSAID}\;\text{intake}\;=\;\text{NSAID}\;\text{equivalent}\;\text{score}\;*\\ \;\;\times \;\frac{\text{Days of intake during period of interest}{\;}^{\wedge }\;\times \;\text{Days per week}}{\text{Period of interest in days}{\;}^{\wedge }} \end{array}$

$$\begin{array}{c} \text{NSAID equivalent score}\;=\;\text{current NSAID dose/optimal dose}\;\times \;100\;\\ \left(\text{If current NSAID dose}\;>\;\text{optimal NSAID dose, then NSAID equivalent score}\;=\;100\right) \end{array}$$

$${\;}^{\wedge }\text{Days}\;\text{of}\;\text{intake}\;\text{during}\;\text{period}\;\text{of}\;\text{interest}\;=\;\text{Period}\;\text{of}\;\text{interest}\;\text{in}\;\text{days}\;=\;30\;\text{days}$$

### 2.4 Disease activity: Bath Ankylosing Spondylitis Disease Activity Index (BASDAI)

BASDAI is a self‐administered questionnaire to assess disease activity using a visual analog scale (VAS). It consists of six questions on fatigue, spinal pain, joint pain/swelling, areas of localized tenderness, and morning stiffness. Scores of each question ranged from 0 (no problem) to 10 (very severe). Further, the mean of BASDAI scores was calculated as follows:

$$\left\{\begin{array}{c} \left(\text{Fatigue score}\right)\;+\;\left(\text{Spinal pain score}\right)\;+\;\left(\text{Joint pain or Swelling score}\right)\;+\;\\ \left(\text{Areas of localized tenderness score}\right)\;+\;\left(\text{Morining stiffness score}\right) \end{array}\right\}/5$$

Further, control level was divided into 2 groups: Optimal control = (BASDAI score < 4), and Sub‐optimal control = (BASDAI score ≥ 4).^26^

### 2.5 QoL: EuroQol‐3L

QoL was assessed using EQ‐5D‐3L, which includes 2 parts; EQ‐5D and the EQ‐VAS.^27^ EQ‐5D consists of the following five domains: mobility, self‐care, anxiety/depression, usual activities, and pain/discomfort, with three levels of responses (no problems, some problems, and extreme problems). EQ‐5D scores range from −0.229 (possible score of the worst imaginable health status) to 1 (possible score of the best imaginable health status) based on a study by Kang et al.^28^ EQ‐VAS addresses self‐rated health on a vertical VAS with scores ranging from 0 (the worst imaginable health status) to 100 (the best imaginable health status). Higher scores of both measurements correspond to a better QoL.

### 2.6 Statistical analysis

For descriptive statistics, continuous variables are presented with the number of observations, the mean, and the standard deviation; categorical variables are presented with frequency and percentage (100%). Chi‐square test, Fisher's exact test and analysis of variance were performed to assess the difference of GI‐related symptoms, NSAIDs adherence, disease activity and QoL depending on the degree of GI risk.

### 2.7 Path analysis model

**Figure 1:**
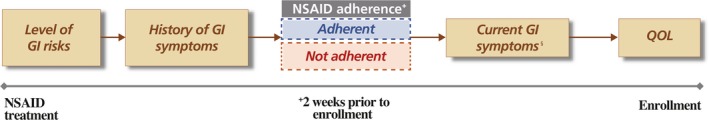
Hypothesized paths of potential determinants to quality of life.* This figure describes hypothesized pathway which was drawn based on the time point of each variable collected and a hypothesized inter‐related association of each variable. ^+^NSAID adherence according to Morisky Medication Adherence Scale‐8 (MMAS‐8) was for the last 2 weeks prior to enrollment. ^§^Timeframe of current GI symptoms and QoL were about the same, but current symptoms were set in the path prior to QoL based on previous study findings showing that current symptoms affected QoL. Abbreviations: GI, gastrointestinal; NSAID, non‐steroidal anti‐inflammatory drug; QoL, quality of life

A hypothetical path model (Figure 1) was drawn based on the specific time point of each variable. The variables included in the hypothetical path model were assumed to be present and/or have impact on the patients from the time point. Also, the inter‐related associations of each variable were considered. The degree of GI risk which took inherent conditions such as age, RA diagnosis, corticosteroid use within 1 year, and history of GI‐related complications into calculation, was placed at the beginning of the path model. The history of GI‐related symptoms that occurred while on NSAIDs treatment was put in the second place since it was considered as a GI risk‐induced factor. The path model was followed by NSAIDs adherence according to MMAS‐8, which was measured during approximately 2 weeks from the study enrollment. Finally, current GI‐related symptoms and QoL were the last factors, but current GI‐related symptoms were set in the path prior to QoL based on logical thinking. In addition to direct paths drawn between each variable in order, based on literature review, we added 2 more direct paths from GI risk and the history of GI‐related symptoms to current GI‐related symptoms and QoL. To perform the path analyses, patients were stratified by control level of disease activity according to BASDAI.

The magnitude of each path is presented with standardized coefficients. Path was considered significant at *P* < .05. The fitness of the path model was evaluated significant for the following: *P *> .05 for Chi‐square, goodness of fit index (GFI) >0.9, adjusted GFI (AGF) >0.85, comparative fit index (CFI) >0.9, Tucker‐Lewis index (TLI) >0.9, root mean square error of approximation (RMSEA) <0.05, and standardized root mean square residual (SRMR) <0.08. The descriptive and path analyses were performed using the SPSS WIN20.0 and AMOS 19.0 Program, respectively.

## 3 RESULTS

The study included 591 AS patients with a mean age of 38.9 ± 12.6 years. Mean duration of AS was approximately 4.3 years. Of the total, 16.1% were diagnosed with GI‐related diseases and about 2% were hospitalized due to GI‐related diseases and complications. Of the total, about one‐third experienced GI‐related symptoms while on NSAIDs treatment and 11.3% showed GI‐related symptoms upon enrollment (Table 1). A majority of the patients were currently on non‐selective NSAIDs and the dosage of current NSAIDs was predominantly moderate. Approximately, 1 in four patients reported as having taken a corticosteroid within a year (Table 2).

**Table 1:** Baseline characteristics of study subjects^a^

	Total (N = 591)
Age, y, mean (SD)	38.9 (12.6)
Male	485 (82.1)
Time to diagnosis from AS onset, mo, mean (SD)	60.9 (80.1)
AS disease duration, mo, mean (SD)	51.8 (46. 8)
GI‐related symptoms and diseases, multiple answers	95 (16.1)
Diarrhea	10 (1.7)
GI pain	9 (1.5)
GI trouble	13 (2.2)
Upper GI ulcer	16 (2.7)
Gastritis	21 (3.6)
GERD	23 (3.9)
Others	86 (14.6)
Co‐morbid diseases,^b^ multiple answers	296 (50.1)
Current health status	
Very poor	26 (4.4)
Poor	192 (32.5)
Normal	258 (43.7)
Good	108 (18.3)
Very good	7 (1.2)
Current smoking	193 (32.7)
Current drinking	372 (62.9)
Current use of antiplatelets	22 (3.7)
Current use of anti‐coagulants	4 (0.7)
History of *Helicobacter pylori* infection	55 (9.3)
History of GI symptom^c^ during NSAID intake	196 (33.2)
Current GI symptoms^c^	67 (11.3)
Hospitalization due to GI‐related disease and complications while on NSAID intake	11 (1.9)
BASDAI, mean (SD)	3.5 (2.0)
Optimal control, <4	366 (61.9)
Sub‐optimal control, ≥4	225 (38.1)
EQ‐5D, mean (SD)	0.6 (0.3)
EQ‐VAS, mean (SD)	67.4 (19.8)

Abbreviations: SD, standard deviation; AS, ankylosing spondylitis; GI, gastrointestinal; GERD, gastro‐esophageal reflux disease; NSAID, non‐steroidal anti‐inflammatory drug; BASDAI, Bath Ankylosing Spondylitis Disease Activity Index.

a Data are N (%) unless indicated otherwise.

b Co‐morbidities included were hypertension (12.4%), eye disease (11.0%), musculo‐skeletal disease (9.3%), infectious disease (5.2%), liver disease (4.6%), skin and subcutaneous diseases (4.6%), genito‐urinary diseases (4.2%), diabetes (4.1%), respiratory disease (3.6%).

c Heartburn and dyspepsia.

**Table 2:** Patterns of currently prescribed NSAID and other related AS treatment^a^

	Total (N = 591)
NSAID type	
Non‐selective NSAID	431 (72.9)
Selective NSAID	160 (27.1)
Duration of NSAID intake	
<3 mo	118 (20.0)
≥3 mo	473 (80.0)
NSAID dose	
Low (<usual dosage)	153 (25.9)
Moderate (usual dosage)	438 (74.1)
High (>usual dosage)	0 (0.00)
NSAID equivalent score, mean (SD)	87.1 (21.9)
GI protective agent	
H_2_ receptor antagonist	69 (13.4)
PPI	172 (33.5)
Others	273 (53.1)
Corticosteroid use for the past 1 year	
Yes	142 (24.0)
Duration of use	
<1 mo	18 (8.9)
1‐3 mo	45 (22.2)
4‐6 mo	44 (21.7)
7‐10 mo	30 (14.8)
11‐12 mo	66 (32.5)
Other drugs, multiple answers	
Narcotic analgesics	2 (0.4)
Non‐opioid analgesics	191 (38.0)
Conventional DMARDs	263 (52.3)
Biologics	188 (37.4)
Selective serotonin reuptake inhibitor	
Yes	3 (0.5)

Abbreviations: NSAID, non‐steroidal anti‐inflammatory drug; AS, ankylosing spondylitis; GI, gastrointestinal; SD, standard deviation; PPI, proton pump inhibitor; DMARDs, disease‐modifying anti‐rheumatic drugs.

a Data are N (%) unless indicated otherwise.

According to the SCORE criteria, 37.1% of patients showed moderate to high GI risk while the remaining had a low GI risk (Table 3). None of the patients showed high adherence to NSAIDs as per the MMAS‐8. Therefore, 55.3% of patients with moderate adherence were categorized as adherent for further analysis. NSAID intake on an average was 73.0 ± 28.2 (Table 4). BASDAI score was 3.5 ± 2.0 on average, and about 40% of patients showed sub‐optimal control of disease activity. Overall, QoL according to EQ‐5D and EQ‐VAS was 0.6 ± 0.3 and 67.4 ± 19.8, respectively (Table 1).

**Table 3:** Assessment of GI risk using Standardized Calculator of Risk for Events (SCORE)

	Total, N = 591 (%)
Low (<10)	372 (62.9)
Moderate (11‐15)	158 (26.7)
High (16‐20)	53 (9.0)
Very high (>20)	8 (1.4)

Abbreviation: GI, gastrointestinal

**Table 4:** Medication adherence of NSAID using MMAS‐8 and NSAID intake rate

	Total (N = 591)	
MMAS‐8^a^	N	589
	Missing	2
	Mean (SD)	5.7 (1.3)
	Adherent,^b^ n (%)	326 (55.2)
	Non‐adherent, n (%)	263 (44.5)
NSAID intake rate	N	586
	Missing	5
	Mean (SD)	73.0 (28.2)

Abbreviations: MMAS‐8, Morisky Medication Adherence Scale‐8; NSAID, non‐steroidal anti‐inflammatory drug.

a MMAS‐8: Adherence was defined if patients showed high or moderate adherence.

b In this study, there was no patient indicating high adherence. Therefore only moderately adherent patients were defined as adherent.

**Figure 2:**
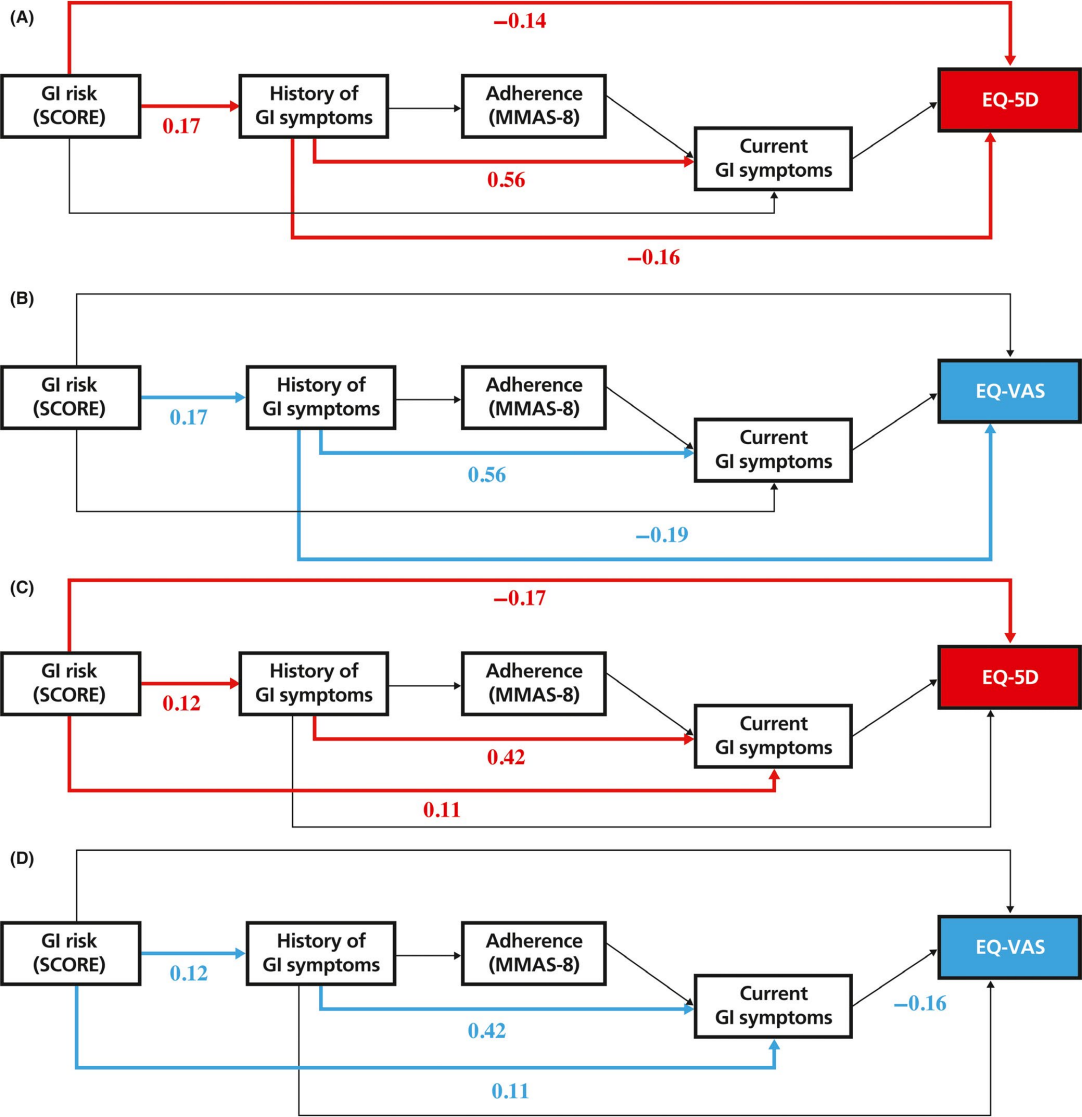
Paths analysis results to quality of life.* A, Pathways of EQ‐5D among patients showing sub‐optimal control on disease activity (BASDAI ≥ 4), N = 225. Goodness of fit: Chi‐square = 12.481 (4 *df*), *P* = .014, GFI = 0.991, AGFI = 0.936, CFI = 0.933, TLI = 0.664, RMSEA = 0.060 (*P* = .277), SRMR = 0.0525. B, Pathways of EQ‐VAS among patients showing sub‐optimal control on disease activity (BASDAI ≥ 4), N = 224. Goodness of fit: Chi‐square = 13.923 (4 *df*), *P* = .008, GFI = 0.990, AGFI = 0.929, CFI = 0.918, TLI = 0.588, RMSEA = 0.065 (*P* = .210), SRMR = 0.0597. C, Pathways of EQ‐5D among patients showing optimal disease control on disease activity (BASDAI < 4), N = 364. Goodness of fit: Chi‐square = 12.481 (4 *df*), *P* = .014, GFI = 0.991, AGFI = 0.936, CFI = 0.933, TLI = 0.664, RMSEA = 0.060 (*P* = .277), SRMR = 0.0908. D, Pathways of EQ‐VAS among patients showing optimal disease control on disease activity (BASDAI < 4), N = 364. Goodness of fit: Chi‐square = 13.923 (4 *df*), *P* = .008, GFI = 0.990, AGFI = 0.929, CFI = 0.918, TLI = 0.588, RMSEA = 0.065 (*P* = .210), SRMR = 0.0889. *Numbers in figures are standardized coefficient. Bold paths indicate statistical significance at *P* < .05. Abbreviations: AGFI, adjusted goodness of fit index; BASDAI, Bath Ankylosing Spondylitis Disease Activity Index; CFI, comparative fit index; GFI, goodness of fit index; GI, gastrointestinal; SCORE, Standardized Calculator of Risk for Events; MMAS‐8, Morisky Medication Adherence Scale‐8; RMSEA, root mean square error of approximation; SRMR, standardized root mean square residual; TLI, Tucker‐Lewis index

In the sub‐optimal control group of patients, higher GI risk significantly lowered EQ‐5D in both direct and indirect ways. Higher GI risk directly impaired the EQ‐5D (b = −0.14, *P* = .015), and it also negatively affected the EQ‐5D through history of GI‐related symptoms (Figure 2A). For EQ‐VAS, GI risk only showed indirect effect on EQ‐VAS which was mediated by the history of GI‐related symptoms (Figure 2B). Likewise, the optimal control group of patients indicated similar trends as that of the sub‐optimal group. Higher GI risk had a direct effect on the decrease in EQ‐5D (b = −0.17, *P* = .014) whereas it was found to have indirect effects on EQ‐VAS through GI‐related symptoms (Figure 2C,2).

## 4 DISCUSSION

We investigated the degree of GI risk and PROs in a large number of AS patients in Korea. The assessment of GI risk among NSAID users has been widely studied using a variety of risk measurement tools in multiple populations. However, only a few studies have enabled the quantification of the degree of GI risk. One cross‐sectional study in Korea, which also applied the SCORE criteria to orthopedic outpatients on NSAIDs, reported about 80% of patients showed higher than moderate GI risk.^10^ In comparison, a lower percentage of our study patients indicated having higher than moderate GI risk. However, as per this study's results, the prevalence of GI risk was noticeable among AS patients; importance of thorough monitoring and management of GI risks and symptoms in AS patients taking NSAIDs warrants attention.

The primary goal of AS treatment is to minimize structural deformity in the spine by preventing joint inflammation and damage. Although no complete cure has been developed to inhibit bone‐bridging and syndesmophytes in the spine, NSAIDs are recommended to relieve symptomatic pain and joint inflammation.^2^, ^29^, ^30^ A randomized controlled trial demonstrated that the continuous use of NSAIDs over 2 years delayed radiographic progression, which was determined as an Modified Stoke Ankylosing Spondylitis Spinal Score (mSASSS) worsening, in the comparison of on‐demand treatment.^31^ In addition, Poddubnyy et al observed a dose‐response relationship between a higher dose of NSAIDs and lower radiographic spinal progression.^32^ Based on these findings, continuous NSAIDs intake, preferably high dose unless contraindicated, is crucial for better management of AS. For these reasons, our study results on NSAIDs adherence was somewhat striking because no patient showed high adherence. To the best of our knowledge, this study might be the first approach to investigate NSAIDs adherence in AS patients since 1996 in which an electronic device was utilized to assess NSAIDs adherence.^13^ Although our results on NSAIDs adherence were merely based on patients’ self‐reports, which may have led to over/under‐estimation of adherence level, the study findings imply that motivational education should be sought to improve NSAIDs adherence which may consequently affect long‐term progression of the disease as well as relief of the symptoms.

The BASDAI has long been studied across countries to assess severity of diseases. In a single‐center study in China, BASDAI of the Chinese patients was similar to that of our findings,^33^ whereas another Asian study involving a total of 85 Korean patients with AS reported less severity than our study patients.^34^ Compared to a previous study of AS patients, our study indicated higher impairment in EQ‐5D.^35^ Although clinical monitoring of AS may be well‐managed, as predicted with their regular visits to university‐based hospitals in metropolitan areas, patient‐perceived disease outcomes might be neglected. Consequently, efforts including the implementation of strategic interventions to enhance QoL need to be introduced from this perspective.

There have been many studies to determine factors associated with QoL in AS patients, with a couple of studies showing that severe disease activity in BASDAI was the key modifiable and a highly associated variable with impairment in QoL.^36^, ^37^ Based on these findings, we divided our study patients into 2 groups being optimal disease activity and sub‐optimal disease activity, and further analyzed the associations of QoL with its potential determinants, the degree of GI risk, the experience of GI symptoms, and NSAIDs adherence using path analysis. With this advanced technique, namely path analysis, we observed that severe GI risk was related to poor QoL, which was mediated by the experience of GI symptoms. We found both direct and indirect pathways of GI risk linked to poor QoL, although the direct paths linking NSAIDs adherence and QoL were insignificant. Due to the nature of this study where cross‐sectional patient survey and retrospective chart review were the primary data sources, it is unclear to infer precise temporal relationships between GI symptoms and NSAIDs adherence.

Although our study included a large number of AS patients who can be a representative of the whole AS population in Korea, the interpretation and utilization of the study results should be carefully done. First, our study utilized both disease‐specific functional disability and generic QoL instruments to measure different aspects of PROs, so the results from the generic QoL instrument were not precisely describing disease‐specific QoL. Our study population may have had better access to hospital care which may have led to better disease management. Also, no direct causal inference may be done due to the nature of the study design. For the assessment of a more accurate and direct causal relationship, more controlled studies should be conducted. Moreover, the study being multi‐centric in nature, clustering has not been taken into account. Intra‐class correlations have not been estimated and reviewed for each variable in the analysis. In addition, recall bias may be present since the assessment on the symptoms/complaints during NSAID usage was based on patients’ self‐reports. Lastly, the interpretation of GI risk, which was assessed by SCORE, should be cautiously done since SCORE is a disease‐specific tool to assess GI risk for arthritis patients, not specific AS patients.

Although our study inherently had several limitations and no direct, causal inference can be made in the relationship of GI risk with QoL, the results clearly provide practical evidence to suggest timely therapeutic strategies be implemented in order to manage GI risk during NSAIDs treatment in AS patients. Therefore, GI risk should be monitored and considered as one of the key factors in order to manage QoL in AS patients on NSAID treatment given there was a significant association between GI risk and QoL. In addition, based on our study objectives and results, we justify the need of a prospective study.

## CONFLICT OF INTEREST

All authors state there is no conflict of interests to disclose.
